# Global child and adolescent mental health perspectives: bringing change locally, while thinking globally

**DOI:** 10.1186/s13034-022-00512-8

**Published:** 2022-11-07

**Authors:** Sowmyashree Mayur Kaku, Jordan Sibeoni, Salah Basheer, Jane Pei-Chen Chang, Dulangi Maneksha Amerasinghe Dahanayake, Matias Irarrazaval, Jamie M Lachman, Boladale Moyosore Mapayi, Anilena Mejia, Massimiliano Orri, Tze Jui-Goh, Md Saleh Uddin, Inge Vallance

**Affiliations:** 1 Centre for Advanced Research and Excellence in Autism and Developmental Disorders (CAREADD), St. John’s Medical College Hospital and St. John’s Research Institute, Bangalore, India; 2 Service Universitaire de Psychiatrie de l’Adolescent, Argenteuil Hopital Centre, Cedex, France; 3 ECSTRRA Team, UMR-1153, Université de Paris, Inserm, Paris, France; 4 Department of Psychiatry, Iqraa International Hospital and Research Centre, Kozhikode, Kerala India; 5 Child Psychiatry Division, Department of Psychiatry, College of Medicine, China Medical University Hospital, Taichung, Taiwan; 6 Department of Psychiatry, Faculty of Medicine, University of Colombo, Colombo, Sri Lanka; 7 Millenium Institute for Research in Depression and Personality, Santiago, Chile; 8 Department of Social Policy and Intervention, University of Oxford, Oxford, UK; 9 Mental health, Obafemi Awolowo University, Ile Ife, Osun State Nigeria; 10 Division of Psychology of Mental Health, The University of Manchester, Manchester, UK; 11 Department of Psychiatry, McGill Group for Suicide Studies, Douglas Mental Health University Institute, McGill University, Montreal, QC Canada; 12 Department of Developmental Psychiatry, Institute of Mental Health, Buangkok Green, Singapore; 13 Jeffrey Cheah School of Medicine and Health Sciences, Monash University (Malaysia campus), Bandar Sunway, Malaysia

**Keywords:** Global Mental Health, Child and Adolescent Mental Health, Child and Adolescent Psychiatry, Communities, Services, Child Mental Health Research, Child Mental Health Policies, Mental Health Professionals

## Abstract

Child and adolescent mental health (CAMH) are a global priority. Different countries across the globe face unique challenges in CAMH services that are specific to them. However, there are multiple issues that are also similar across countries. These issues have been presented in this commentary from the lens of early career CAMH professionals who are alumni of the Donald J Cohen Fellowship program of the IACAPAP. We also present recommendations that can be implemented locally, namely, how promoting mental health and development of children and adolescents can result in better awareness and interventions, the need to improve quality of care and access to care, use of technology to advance research and practices in CAMH, and how investing in research can secure and support CAMH professionals and benefit children and adolescents across the globe. As we continue to navigate significant uncertainty due to dynamic circumstances globally, bolstering collaborations by “bringing change locally, while thinking globally” are invaluable to advancing global CAMH research, clinical service provision, and advancement of the field.

**Supplementary Information:**

The online version contains supplementary material available at 10.1186/s13034-022-00512-8.

## Introduction

Child and adolescent mental health (CAMH) is a global priority. The World Health Organisation (WHO) supports “development and implementation of multisectoral, evidence-informed and human-rights-based strategies for the promotion of mental health, prevention of mental health conditions and provision of mental health care for children, adolescents and their families” [1]. Every endeavor towards improving CAMH services and research should be country-specific but should contain the best evidence from across the globe [2]. There is a drive towards improving global health and a slight but not enough momentum in the areas of CAMH and allied specialties [3, 4].

The International Association for Child and Adolescent Psychiatry and Allied Professions (IACAPAP) which brings together, professionals working across the globe in areas of CAMH, states its mission as – “to advocate for the promotion of the mental health and development of children and adolescents through policy, practice, and research” [5]. The IACAPAP has mentoring programs such as the *Donald J. Cohen Fellowship Program for International Scholars in Child and Adolescent Mental Health and Helmut Remschmidt Research Seminars* for young professionals in CAMH. These awards help foster the professional development of emerging leaders and inspire young CAMH colleagues to engage in CAMH research, respectively [5].

Aims and methods:

We, CAMH professionals who were participants of the Donald J Cohen Fellowship Program (DJCFP) at various times in the last decade (Range: 2010–2020, Median: 2014), contributed to this manuscript. Authors are from the backgrounds of adult psychiatry, child and adolescent psychiatry, public health, clinical psychology, neurosciences, prevention and implementation science and several have multi-country work experiences. The DJCFP program helped bolster discussions about the global nature of CAMH and made an impact on all of us. The enriching experience got us thinking about similarities and differences in CAMH scenarios in our respective countries and brought insights on how we can bring about change at the local stage while thinking globally. This paper aims to contribute further to these discussions by presenting CAMH perspectives from 11 countries (Bangladesh, Chile, France, India, Italy, Nigeria, Panama, Singapore, South Africa, Sri Lanka, and Taiwan), reviewing how they can influence global CAMH and presenting the way forward. These are the home countries of authors where they studied and trained in CAMH. Though we are presenting perspectives from countries that are diverse, we believe that this weakness is overcome by the ideas we present for all countries to engage with one another and set up collaborative care and research. The content of this commentary was derived from write-ups from each author’s perspective, to answer common questions such as prevalence, awareness, resources, clinical services and research, barriers to implementations, manpower training, policy initiatives, and experiences as early-career professionals. These write-ups were collated to identify common issues, key take aways, potential recommendations and the way forward in CAMH.

## Current CAMH scenarios across 11 countries

### African countries

- Nigeria

In Nigeria, over the last few years, there has been minimal but increased awareness on CAMH issues. There is an enormous shortage of CAMH practitioners [6] made worse by highly skilled professionals leaving the country. Though more has been done to improve services for CAMH, the country still needs to do more in organizing services for CAMH. There is a need for national surveys on the burden of child mental health problems to encourage advocacy and increase funding for CAMH service provision.

- South Africa

Data on the prevalence of CAMH problems in South Africa is limited, but findings from an epidemiological study from one of the nine provinces found the overall prevalence to be at 17%, with the most common problem being generalized anxiety disorder in adolescence [7]. Considering that almost 40% of the country’s population is under 18 years of age, this figure translates into a significant public health burden.

### European countries

- France

In France, epidemiological data in CAMH are scarce. Data from INSERM (national institute of medical health research) indicates that one in eight have mental health disorders, 5% of children under 12 suffer from anxiety disorders, 1–2% attention deficit hyperactivity disorders (ADHD), and 0.5% depression. In adolescence, mood disorders affect 3% of 13–19-year-old [8]. Mental health issues are better addressed through earlier and more accurate diagnosis and increased recognition. There is, in fact, better awareness in the French population regarding the mental health needs of children and a greater societal demand with more and more families seeking professional help on various topics, including ADHD, learning disorders, Autism Spectrum Disorder (ASD), school refusal, bullying, and eating disorders.

- Italy

The prevalence of child and adolescent mental disorders in Italy is estimated at around 8% (presence of at least 1 Diagnostic Statistical Manual diagnosis, 10–14 years) [9]. This is comparable with other European regions, although lower for specific conditions (e.g., Italy has a low suicidal behavior rate). CAMH services are however responsible for the assessment, and treatment of both psychiatric and neurological conditions.

### Asian countries

- Singapore

In Singapore, there is no prevalence study on the estimates of CAMH disorders, although studies on adults and the elderly suggest childhood-onset of several conditions [10]. One community-based study examining mental health issues in school-going children between 6 and 12 years old reported a comparable prevalence figure of 12.5%, with more internalizing problems than externalizing problems being reported by parents[11]. Economically, ASD is on the top in terms of disease burden followed by ADHD and depressive disorders for children under the age of 14 [12]. An area of increasing concern pertains to the use of the internet and social media among adolescents. A study estimated the prevalence of pathological gaming to be 8.7% [13], but this figure is merely the tip of an iceberg given that the impact of technology use is not fully understood currently, but one would postulate an increased risk for the development of mental and physical health concerns.

- Taiwan

The Prevalence rate of psychiatric disorders in children and adolescents in Taiwan ranges from 14.8 to 22.7%. For example, the prevalence rate of ADHD is around 7.5% in Taiwan [14].

- Bangladesh

The prevalence of mental disorders in Bangladesh is 13.4 to 22.9% among children and adolescents [15, 16]. Government expenses 0.5% of the total health budget in mental health. There are ongoing efforts to expand training and research in CAMH.

- India

Though quite variable in many studies, a systematic review reported the prevalence rate of child and adolescent psychiatric disorders in the community to be 6.46% [17]. Efforts such as setting up child guidance clinics and setting up specialized fellowship training did help to improve the infrastructure and expertise. However, only 0.06% of the total national health budget is allocated to mental health and a small portion of this tiny budget is given for CAMH services. Such low budgetary allocation that is lower than most low- and middle-income countries (LMIC) has resulted in specialized care being difficult to access and limited. It is estimated that < 1% of the children and adolescents suffering from mental disorders receive treatment [18].

- Sri Lanka

Sri Lanka has an established network for maternal and child health services governed by a national policy [19]. However, the development of child psychiatric services lags with limited research in this population. A nationwide survey estimated that 18.9% of children aged 13–19 years have significant emotional and behavioral issues and another study estimated the prevalence of ASD to be 1:93 [20, 21]. Evolving socio-cultural changes have impacted family systems, education, values, and mental health needs.

### South american countries

- Chile

In Chile, the general prevalence of mental disorders in children and adolescents is 22.5%, higher in females (25.8% compared to 19.3% in males), and in children aged 4–11 years (27.8%) compared to adolescents (16.5%)[22]. Compared to other international epidemiological studies these numbers show that mental disorders in children and adolescents, alcohol and drug abuse and suicide rates in adolescents have a higher prevalence in Chile compared to the rest of the world. These epidemiological findings reflect the inequalities that are present in Chile, the most unequal country among the Organization for Economic Cooperation and Development group [23].

- Panama

Research on CAMH in Panama is scarce. A study conducted in 2009 suggested that the most prevalent disorders in primary school children (6–12 years old) are learning difficulties (20.6%) and anxiety (15.3%). The prevalence of depression is 5.3%. This same study suggested that only one in ten children with a disorder has had contact with mental health services. Low access to mental health services has to do with a lack of investment and trained professionals in public services to cope with the demand of cases. Only 3% of the Ministry of Health’s total budget is directed towards mental health [24]. There are 3.47 psychiatrists per 100,000 inhabitants and only 2 outpatient facilities for children and adolescents in the whole country, considering that 33% of users are children. Given the high rate of disorders, preventive programs are becoming more popular.

## Common issues across the globe

### Armed conflicts and social unrest:

Many children in low- and high-income countries with ongoing armed conflicts and social unrest are frequently exposed to poor physical, mental, and social conditions that impact their emotional development. It exposes them to violence, lack of education, illnesses, torture and sexual violence, displacement, social isolation, discrimination, and many times poor long-term prognosis due to anxiety, depression, post-traumatic stress disorder (PTSD), and substance abuse. Striking examples include incidents in Nigeria (the Chibok girls), France, India (terrorism) and war (Sri Lanka, India and even the ongoing Russian-Ukraine war) in many parts of the world.

### Poverty and social inequalities

The focus of child healthcare in most LMIC countries has been on the childhood ‘killer’ diseases such as diarrhoea, acute respiratory infections, and malaria almost excluding the mental aspect of child health [2]. Specific risk factors include widespread family poverty and social inequalities (based on caste, color, ethnicity, and religion).

Studies have shown links between family poverty and the risk of child neglect [3, 4]. Poverty often leads to malnutrition, deprivation of neighborhood and educational opportunities, and poor parental mental health. Millions of children and adolescents are still growing up in circumstances of significant adversity. Many are exposed to substance abuse and violence as well as HIV infection and risk for orphanhood – all of which increase vulnerability to mental health problems (like in South Africa) [25]. For example, children orphaned by AIDS have higher levels of depression, anxiety, and PTSD when compared to other orphaned and non-orphaned children [26].

Many high-income countries are fighting societal inequalities that impact childhood development, social bonding, sense of autonomy, independence, self-esteem, and self-confidence [27, 28]. Racism, religion, and caste system-based factors impact access to health services, educational and economic opportunities [29, 30]. They also impact brain development and result in poor psychological development due to chronic stress [28, 31].

### Child guidelines and policies

Though national policies are in place in many countries, the implementation of the national plans are hindered by the unavailability of provincial-level resources or policies [10, 25, 32–35]. Most countries still focus on maternal care, immunization, nutritional support, and early education. In the majority of cases, there is a lack of ‘political will’ since the focus is mainly on mortality data from maternal and child health. There is also a lack of resources to formulate, motivate or co-ordinate the implementation of child mental health programs [7, 36, 37]. In those countries where they do exist, these programs have a plethora of roadblocks at the levels of prevention services, budget allocation and other priorities in childcare service pathways.

### Clinical services

The health system of most countries is severely constrained, with an estimated < 0.5 psychiatrists per 100,000 population [2, 17, 38–40]. This translates into just a small fraction of children and adolescents accessing needed CAMH services, thus requiring transfer of skills and task sharing between professionals and community health workers, and traditional healers [37]. The territorial distribution of CAMH professionals is also marked by strong disparities in many countries.

CAMHS are responsible for the care of patients up to 17 years, and once 18 years old, patients are referred to adult services. Literature suggests that around one in five adolescents in contact with CAMHS moved to adult services, pointing out gaps in the continuity of care for youths across local services [41].

Most countries do have a national association or society for child and adolescent mental health that are members of the IACAPAP and have been trying to build a platform for child mental health professionals.

### Funding and research for early career professionals

Difficulties specifically encountered by early-career CAMH professionals include limited funding and inequalities in research opportunities [42–45]. As many governments do not prioritize child mental health research or have allocated budget in their ‘areas of interest’, research in CAMH is predominantly restricted to popular university hospitals, experienced and well-established researchers, and their students. When professionals transition to become independent researchers outside of these settings, the opportunities are almost nil, thus encouraging the vicious cycle of brain drain to the global North. Most non-university departments, child guidance clinics, and non-governmental organizations do not have either resources or means to do research.

### Work-life integration and well-being

Work-life integration is a challenge for early career professionals in most countries. Early career CAMH professionals, like all other specialties, typically are settling down with their families and often have young children. The heavy workload due to lack of specialized resources, poor work schedules, limited leaves, lack of funds and facilities for childcare and self-care add to the burden [46–48]. Lack of mentors to support, and facilities that address these challenges also add to the poor mental health of early career professionals [49].

### Way forward

Based on our experiences as professionals working in CAMH, we offer recommendations that are an offshoot of the global challenges discussed above. Bolstering local initiatives, improvising strategies to address CAMH, and supporting professionals at the grassroot level, can empower and improve services and research. We discuss these in four sections that over arch the common issues across the globe, while understanding the need to have common solutions to work at our home countries.

#### *Promoting mental health and development of children and adolescents*

More and more countries are turning into “Social Investment Welfare States” aiming to invest in the potential of children today to ensure the welfare state tomorrow. This requires the implementation of comprehensive national policies to promote the mental health and development of children and adolescents [16, 33, 34, 50–52]. These policies adopt a holistic view combining children’s social protection and CAMH.

Yet, some CAMH professionals reported spontaneously that in their country, mental health promotion programs rely mostly on local initiatives despite national mental health guidelines or policies. E.g., India’s recent Mental Healthcare Act 2017 recognizing the special needs of young people [50] period In Bangladesh, national mental health policy included school mental health programs that recommended the inclusion of mental health curriculum in science text books [53].

#### *Fighting stigma through literacy*

Improving literacy levels of the population is a global challenge to fight the stigma around mental illness and solve the problem of underutilization of mental health services [54]. Five CAMH professionals reported that in their country the information on childhood psychiatric disorders such as Intellectual Disability, ADHD, and ASD are still limited to the general public which results in stigma, hence more effort needs to be spent on psychoeducation programs [55–59].

#### *Community-level interventions*

Many countries are investing in early childhood interventions such as parenting or psychoeducation programs and community-level (very often school-based) interventions in their country. Indeed, school-based interventions are less stigmatizing and are effective in promoting mental health, including in LMIC settings [36, 53]. It is also important to reinforce collaborative care between CAMH professionals and health specialists or physicians in the community while upscaling skill sets of allied health professionals, community nurses and educators to screen, identify and manage CAMH disorders. This would help address the shortage of professionals and constitute to a stepped-care model which is currently being adopted by many countries.

#### *Targeting non-mainstream problems*

When discussing CAMH, as part of the holistic approach, interventions and research in relevant areas such as child abuse, maltreatment, the mental health of orphan and street children, children in conflict with the law, have to be taken into consideration as these can have long-lasting mental and physical health benefits that extend into later adolescence and adulthood [60–64]. This particularly highlights factors influencing CAMH that are global and emphasizes that it should be approached as such.

#### Improving access to quality care

Lack of access to specialized CAMH care or important delay due to the shortage of CAMH professionals is a shared concern across the globe. More funding, more training, more valorisation, and more resources are needed to improve both access and quality of CAMH care.

While many countries have ongoing training programs, some countries have developed specific programs focusing on training professionals so to increase manpower in CAMH services. Example:

- In Nigeria, the inception of the Centre for Child and Adolescent Mental Health at the University of Ibadan - with funding from the John D. and Catherine T. MacArthur Foundation has led to increased manpower training [6, 65].
- In Singapore, even if the number of child psychiatrists remained small, the number of allied services has flourished exponentially (both specialized services -e.g., Neuro-Behavioural Clinic for neurodevelopmental disorders- and community teams) [39].
- A revalorization of the Child and Adolescent Psychiatry specialty appear to be also necessary, especially among countries with an aging population [14].

#### *Accurate and adequate clinical practice*

Adopting a culturally sensitive approach and developing interventions targeting uncovered/ insufficiently covered issues would help develop and improve the quality of CAMH services [66–68].For instance, in France, the recent terrorist attacks raised many concerns about post-traumatic stress disorder and led to the creation of the CN2R (Centre national de resource et resilience) to deepen and improve knowledge about PTSD and resilience [69].

#### *Expanding the use of technology*

Embracing innovation and advances in technology to improve prevention and mental health outcomes can benefit children and adolescents globally. In many countries:, technology is increasingly being used in awareness campaigns, screening patient consultations, improving follow-up, training of professionals, and research [70, 71]. Technology can also be used to achieve population-level benefits and enhance efforts at task shifting, for instance using digital mental healthcare model tool to deliver quality psychiatric care by non-specialists in remote areas [72].

Many research laboratories all over the globe are incorporating new technologies in areas such as artificial intelligence, machine learning, and robotics into child and adolescent psychiatric care, especially neurodevelopmental disorders, thus facilitating early identification and early intervention [73, 74].

In the past two years of the COVID-19 pandemic, CAMH services in many countries have been developing teleconsultations, online interventions and self-directed resources for children and their parents as well [75–79].

#### *Investing in Research*

Research in CAMH is gradually increasing worldwide, from interests in basic neuroscience, translational psychiatry, research in treatment approaches, etiology and clinical trials. Many countries have state-funded and private centres of excellence for ASD and neurodevelopmental disorders. However, lack of resources and research funding remains the main obstacle, especially for LMIC countries but also in high-income countries like Italy where there is a lack of time dedicated to doing research for clinicians. Early career CAMH professionals often do research as secondary activity aside from the clinical work and only a few envision a career in pure research.

There is a need for research to focus on establishing the efficacy and cost-effectiveness of care and services. Last but not the least, the role of patients and their parents in their medical management radically evolves. More collaborative practices, that consider the patient’s perspective in this process, are progressively replacing paternalistic medicine. Qualitative research is in line with this societal evolution and is booming in biomedical clinical studies. For example, in France, Anne Revah-Levy’s research group has developed expertise in their use for exploring complex questions around the lived experience of mental disorders and their treatment among children and adolescents [80].

## Conclusion

The DJCFP gave us a platform to come together, maintain these network connections, and discuss and inspire each other in what we do, which we believe is a return on investment of the fellowship program itself. The alumni have also been in leadership roles across various national and global organizations, organizing meetings in CAMH themes and even volunteering at the forefront of war and pandemics.

In summary, though there are differences in treatment models, prevalence rates, availability of resources, and cultural practices, we believe the challenges we face are similar. The need to address armed conflicts, social unrest, social inequalities, pushing for child mental health guidelines and policies, and improving the quality of clinical care and services cannot be emphasized enough. Strategies to improve research funding, with a focus on early career CAMH professionals, opportunities for work-life integration along with appropriate use of manpower and technology will go a long way. We conclude by highlighting that bringing change locally while thinking globally will undoubtedly bolster CAMH.

## Electronic supplementary material

Below is the link to the electronic supplementary material.

Supplementary Material 1

Supplementary Material 2

## Data Availability

Not applicable.
