# Supplementary material for: Global child and adolescent mental health perspectives: bringing change locally, while thinking globally
Source: Child Adolesc Psychiatry Ment Health. 2022 Nov 7;16:82. doi: 10.1186/s13034-022-00512-8 (PMC9640779; doi:10.1186/s13034-022-00512-8)
Supplement: Supplementary file 1 — Supplementary Material 1 [file 13034_2022_512_MOESM1_ESM.docx]

| Country | Population demographics of children and adolescents |
| --- | --- |
| Nigeria | Nigeria has a population of over 200 million, and over half of these are children and adolescents. |
| South Africa | South Africa is an upper-middle-income country with a population of 57.78 million people and one of the highest levels of inequality in the world. 40% of the population are under 18 years of age. |
| France | Children and adolescents make up 18% of the population of France. |
| Italy | Italy has about 10 million children and adolescents. |
| Singapore | Singapore is a young city-state with a population of about 5.5 million, with about 16.6% of the resident population under the age of 20 years. |
| Bangladesh | Bangladesh is a country of almost 161 million people. 33 million children adolescents live in poverty. |
| India | India has more than 434 million children and adolescents, probably the highest in the world. |
| Sri Lanka | Sri Lanka has more than 5 million children aged below 15 years. |
| Taiwan | Children between 0-14 years of age make up 12% of the population of Taiwan. |
| Chile | Children and adolescents are 21% of Chile’s 19 million population. |
| Panama | Children and adolescents are 28% of Panama’s 4.5 million population. |
