# Supplementary material for: Global child and adolescent mental health perspectives: bringing change locally, while thinking globally
Source: Child Adolesc Psychiatry Ment Health. 2022 Nov 7;16:82. doi: 10.1186/s13034-022-00512-8 (PMC9640779; doi:10.1186/s13034-022-00512-8)
Supplement: Supplementary file 2 — Supplementary Material 2 [file 13034_2022_512_MOESM2_ESM.docx]

Supplementary table 2: Details of prevalence, mental health programs, policies, and initiatives in various countries

| Country | Details on prevalence, mental health programs, policies, and initiatives as available |
| --- | --- |
| Nigeria | Currently, small-scale epidemiological surveys for child mental health problems have been carried out in Nigeria. There is a need for national surveys on the burden of child mental health problems. The Maternal and Child Health Integrated Program (MCHIP) for under-5 Nigerian children does not have specific guidelines for CAMH (1). Nigeria does not have a CAMH policy since the focus is mainly on mortality data from maternal and child health. There is also lack of resources to formulate, motivate or co-ordinate the implementation of CAMH programs. |
| South Africa | Data on prevalence of CAMH problems in the country is limited, but findings from an epidemiological study from one of the nine provinces found overall prevalence to be at 17%, with the most common problem being generalized anxiety disorder in adolescence (2). Considering that almost 40% of the country’s population is under 18 years of age, this figure translates into a significant public health burden. In terms of CAMH policy, Mokitimi and colleagues (2018) found that while there is a national policy in place, none of the provinces had a publicly-available provincial policy or plan, which hinders the implementation of the national plan (3). Considering challenges with service provision and the substantial burden of CAMH problems, effective and scalable interventions are needed to promote mental health and resilience (4). |
| France | In France, epidemiological data are scarce when it comes to child and adolescent psychiatry. A collective expertise from INSERM (national institute of medical health research) indicates that one child out of eight in France suffers from a mental disorder, 5% of children under 12 suffer from anxiety disorders, 1-2% ADHD, and 0.5% depression. In adolescence, mood disorders affect 3% of 13–19-year-old. Autism spectrum disorders (ASD) and schizophrenia affect less than 1% of children and adolescents. Recent CAMH policies in France focus on diagnostic and treatment of neurodevelopmental disorders(5), prevention and management of suicidal behaviors among children and adolescents (6), and coordination between child welfare services and CAMH services (7). |
| Italy | The prevalence of C&A mental disorder in Italy is estimated at around 8% (presence of at least 1 DSM diagnosis, 10–14 years) (8). The findings of a recent study suggested that around one in five adolescents in contact with CAMHS moved to adult services, pointing out gaps in the continuity of care for youths across local services (9). Steps have already been taken to help those suffering from COVID-19-related stress. The national mental health service was launched in March 2020, to combat rise in mental health issues. This intends to provide free emergency services offered by psychoanalysts and psychologists to all, with particular emphasis on low-income families and individuals living independently. |
| Singapore | No formal or systematic prevalence studies has been conducted in the area of child and adolescent mental health in Singapore although several studies are currently underway or planned (at the time of this writing).  In terms of Years Lived with Disability (YLDs), neurodevelopmental disorders were the second largest contributor (under the Mental Health conditions category) in 2017 (Ministry of Health Singapore, Epidemiology & Disease Control Division & Institute for Health Metrics and Evaluation, 2019). In children, ASD was the leading cause of disease burden, followed by attention deficit hyperactivity disorder (ADHD) in the 3^rd^ position, in Singaporean children aged 0 -14 years(10).  Based on the expenditure data from the Ministry of Health (MOH), about SGD 300 million has been spent on mental health in the financial year 2017. This cost comprises operating subvention to public healthcare institutions and intermediate and long-term care facilities to subsidize patient care as well as funding for programmes under the National Mental Health Blueprint and Community Mental Health Masterplan. The Enabling Masterplans (EMP) was established and is currently in its 3^rd^ iteration. It formed recommendations for the facilitation of services provision, policies and initiatives to improve the lives of people with disabilities, including children and adolescents with neurodevelopmental disorders (11).  In 2007, MOH implemented the National Mental Health Blueprint which supported the introduction of programmes to improve early detection and treatment for persons with mental health conditions. For example, the Response Early Intervention and Assessment in Community Mental Health (REACH) was set up to provide students with emotional, social and behavioural issues within the school and community(12). |
| Bangladesh | The reported prevalence of mental disorders varied from 6.5 to 31.0% among adults and from 13.4 to 22.9% among children (13). The Mental Health Act was passed by the national Parliament in 2018. Mental health policy approved Ministry of health, Bangladesh in 2019 (14). With treatment gaps as large as 92% for adults and 94% for children according to some studies (15). Mental health expenditures by the Bangladeshi government are 0.44% of the total health budget (16). |
| India | Though quite variable in many studies, a systematic review reported the prevalence rate of child and adolescent psychiatric disorders in the community to be 6.46% (17). Efforts such as setting up of child guidance clinics and setting up of specialized fellowship training did help to improve the infrastructure and expertise (18). However, only 0.06% of the total national health budget is allocated to mental health and a small portion of this tiny budget is given for Child and Adolescent Mental Health (CAMH) services. In addition to this inadequate coverage of the CAMH services, there is the added problem of underutilization of all mental health services due to problems like stigma in India (19). Though the new Mental Healthcare Act 2017 is more cognizant of the special needs of young people, India lacks a comprehensive CAMH policy (20). Such a holistic policy is needed to effectively address all aspects of CAMH challenges and to reduce its economic burden on a large country like India. |
| Sri Lanka | There is a scarcity of studies exploring the prevalence of psychiatric disorders among children and adolescents in Sri Lanka. Ginige et al found  a prevalence of 13.8% for behavioural and emotional disorders among schoolchildren between the ages of 7-11 years in the Kandy district in Sri Lanka (21). In 2009, Perera et al found that the prevalence of autism among 18-24 month-year-olds in a semi-urban area in Sri Lanka to be 1.07% (22). The mental health policy of Sri Lanka specifically addresses the importance of developing specialised child and adolescent psychiatry services as well as mother and baby services. However, over the years the emphasis was placed on establishing general psychiatry services to minimise the mental health gap and provide specialist services to all districts of the country.  Currently, specialised child and adolescent psychiatry units are located only in five districts of the country, with general adult psychiatrists providing services in other areas. Many families have difficulties travelling to specialised services and during the COVID pandemic and the economic crisis of the post-COVID era, families continue to struggle to access services. Total current health expenditure of Sri Lanka in 2018 was around Rs. 559,100 million and amounted to 3.9 % of GDP, out of which Rs. 3,192.4 million (0.6%) was allocated to mental (psychiatric) disorders and Rs. 9.3 million to behavioural disorders (23,24). |
| Taiwan | The Prevalence rate of psychiatric disorders in children and adolescents in Taiwan ranges from 14.8% to 22.7%. For example, the prevalence rate of attention deficit hyperactivity disorder (ADHD) is around 7.5% in Taiwan (25). The current research difficulties encountered by early career psychiatrists are that there are limited funding and inequality in research opportunities in the field of child mental health. Some of the issues faced by Taiwanese early career CAPs are that the hospital may underestimate the value of such subspecialty, since Taiwan is becoming an aged country. Moreover, the information on childhood psychiatric disorders such as ADHD and autism are still limited to the general public which result in stigma, hence more effort needs to be spent on psychoeducation programs. |
| Chile | In Chile, the general prevalence of mental disorders in children and adolescents is 22.5%, higher in females (25.8% compared to 19.3% in males), and in children aged 4-11 years (27.8%) compared to adolescents (16.5%) (26). Policy initiatives in Chile's health sector are gradually constructing a system that complies with the UN Convention on the Rights of the Child. Several legislative initiatives to enhance child protection are now on the active parliamentary agenda and a new National Policy on Children and Adolescents has been enacted in 2022. The Gender Identity Bill and the Law of Videotaped Child Interviews Before Courts are two that have been approved. The Mental Health Legislation, an amendment to the Adoption Bill, and a new law for children in protective care or the juvenile justice system are among the others legislative projects that has being advancing (27). |
| Panama | A study conducted in 2009 suggested that the most prevalent disorders in primary school children (6-12 years old) are learning difficulties (20.6%) and anxiety (15.3%). Prevalence of depression is 5.3%. This same study suggested that only one in ten children with a disorder have had contact with mental health services. Low access to mental health services has to do with lack of investment and trained professionals in public services to cope with the demand of cases. Only 3% of the Ministry of Health’s total budget is directed towards mental health(28). |

References:

1. NEWBORN HEALTH in the context of the Integrated Maternal, Newborn and Child Health Strategy Saving Newborn Lives in Nigeria: FEDERAL REPUBLIC OF NIGERIA MINISTRY OF HEALTH 2 SAVING NEWBORN LIVES IN NIGERIA SAVING NEWBORN LIVES IN NIGERIA 3.

2. Kleintjes S, Flisher AJ, Fick M, Railoun A, Lund C, Molteno C, et al. The prevalence of mental disorders among children, adolescents and adults in the Western Cape, South Africa. Afr J Psychiatry. 2006 Oct 2;9(3):157–60.

3. Mokitimi S, Schneider M, de Vries PJ. Child and adolescent mental health policy in South Africa: History, current policy development and implementation, and policy analysis. Int J Ment Health Syst. 2018 Jun 26;12(1):1–15.

4. Mendenhall E, De Silva MJ, Hanlon C, Petersen I, Shidhaye R, Jordans M, et al. Acceptability and feasibility of using non-specialist health workers to deliver mental health care: stakeholder perceptions from the PRIME district sites in Ethiopia, India, Nepal, South Africa, and Uganda. Soc Sci Med. 2014;118(C):33–42.

5. Haute Autorité de Santé - Trouble du neurodéveloppement/ TDAH : Diagnostic et prise en charge des enfants et adolescents - Note de cadrage [Internet]. [cited 2022 Jul 22]. Available from: https://www.has-sante.fr/jcms/p_3302482/fr/trouble-du-neurodeveloppement/-tdah-diagnostic-et-prise-en-charge-des-enfants-et-adolescents-note-de-cadrage

6. Haute Autorité de Santé - Idées et conduites suicidaires chez l’enfant et l’adolescent : prévention, repérage, évaluation et prise en charge [Internet]. [cited 2022 Jul 22]. Available from: https://www.has-sante.fr/jcms/p_3288864/fr/idees-et-conduites-suicidaires-chez-l-enfant-et-l-adolescent-prevention-reperage-evaluation-et-prise-en-charge

7. Haute Autorité de Santé - Coordination entre services de protection de l’enfance et services de pédopsychiatrie - Note de cadrage [Internet]. [cited 2022 Jul 22]. Available from: https://www.has-sante.fr/jcms/p_3261731/fr/coordination-entre-services-de-protection-de-l-enfance-et-services-de-pedopsychiatrie-note-de-cadrage

8. Frigerio A, Rucci P, Goodman R, Ammaniti M, Carlet O, Cavolina P, et al. Prevalence and correlates of mental disorders among adolescents in Italy: the PrISMA study. Eur Child Adolesc Psychiatry 2009 184. 2009 Jan 22;18(4):217–26.

9. Stagi P, Galeotti S, Mimmi S, Starace F, Castagnini AC. Continuity of care from child and adolescent to adult mental health services: evidence from a regional survey in Northern Italy. Eur Child Adolesc Psychiatry 2015 2412. 2015 Jul 4;24(12):1535–41.

10. P PH, V L CA, K CS. Singapore’s burden of disease and injury 2004 Introduction: The Singapore Burden of Disease. Orig Artic Singapore Med J. 2009;50(5):468.

11. 3rd Enabling Masterplan 2017-2021: caring nation, inclusive society - BookSG - National Library Board, Singapore [Internet]. [cited 2022 Jul 22]. Available from: https://eresources.nlb.gov.sg/printheritage/detail/dda160d8-1259-4a94-8923-80d35244041b.aspx

12. Lim CG, Loh H, Renjan V, Tan J, Fung D. Child Community Mental Health Services in Asia Pacific and Singapore’s REACH Model. Brain Sci. 2017 Oct 6;7(10).

13. Hossain MD, Ahmed HU, Chowdhury WA, Niessen LW, Alam DS. Mental disorders in Bangladesh: a systematic review. BMC Psychiatry. 2014 Jul 30;14(1).

14. Bangladesh WHO Special Initiative for Mental Health Situational Assessment I. CONTEXT.

15. Islam A, Biswas T. Mental Health and the Health System in Bangladesh: Situation Analysis of a Neglected Domain. http://www.sciencepublishinggroup.com. 2015;3(4):57.

16. Hasan MT, Anwar T, Christopher E, Hossain S, Hossain MM, Koly KN, et al. The current state of mental healthcare in Bangladesh: part 2 – setting priorities. BJPsych Int. 2021 Nov;18(4):82–5.

17. Malhotra S, Patra BN. Prevalence of child and adolescent psychiatric disorders in India: A systematic review and meta-analysis. Child Adolesc Psychiatry Ment Health. 2014 Jul 21;8(1):1–9.

18. Malhotra S, Vikas A. Pervasive Developmental Disorders: Indian Scene. Vol. 1, Review Article JIACAM.

19. Shidhaye R, Kermode M. Stigma and discrimination as a barrier to mental health service utilization in India. Int Health. 2013 Mar 1;5(1):6–8.

20. Sharma E, Kommu J. Mental Healthcare Act 2017, India: Child and adolescent perspectives. Indian J Psychiatry. 2019 Apr 1;61(Suppl 4):S756–62.

21. Ginige P, Tennakoon SUB, Wijesinghe WHMKJ, Liyanage L, Herath PSD, Bandara K. Prevalence of behavioral and emotional problems among seven to eleven year old children in selected schools in Kandy District, Sri Lanka. J Affect Disord. 2014 Oct 1;167:167–70.

22. Perera H, Wijewardena K, Aluthwelage R. Screening of 18–24-Month-Old Children for Autism in a Semi-Urban Community in Sri Lanka. J Trop Pediatr. 2009 Dec 1;55(6):402–5.

23. pg. Policy Repository-Ministry of Health-Sri Lanka The Mental Health Policy of Sri Lanka Preamble.

24. (3) (PDF) Child and Adolescent Psychiatry in Sri Lanka [Internet]. [cited 2022 Jul 22]. Available from: https://www.researchgate.net/publication/344436122_Child_and_Adolescent_Psychiatry_in_Sri_Lanka?channel=doi&linkId=5f7535e0a6fdcc00864bf6a6&showFulltext=true

25. Gau SSF, Chong MY, Chen THH, Cheng ATA. A 3-year panel study of mental disorders among adolescents in Taiwan. Am J Psychiatry. 2005 Jul 1;162(7):1344–50.

26. Vicente B, Saldivia S, De La Barra F, Kohn R, Pihan R, Valdivia M, et al. Prevalence of child and adolescent mental disorders in Chile: A community epidemiological study. J Child Psychol Psychiatry Allied Discip. 2012 Oct;53(10):1026–35.

27. Ejecutiva S. HACIA UN SISTEMA DE GARANTÍAS DE DERECHOS DE LA NIÑEZ. 2014;

28. WHO-AIMS Report on Mental System on Panama [Internet]. Available from: https://www.who.int/mental_health/evidence/panama_who_aims_report.pdf
